# Diflunisal inhibits prestin by chloride-dependent mechanism

**DOI:** 10.1371/journal.pone.0183046

**Published:** 2017-08-17

**Authors:** Guillaume Duret, Fred A. Pereira, Robert M. Raphael

**Affiliations:** 1 Rice University, Department of Electrical and Computer Engineering, Houston, Texas; 2 Huffington Center on Aging, Department of Otolaryngology–Head and Neck Surgery and Department of Molecular and Cellular Biology, Baylor College of Medicine, Houston, Texas; 3 Rice University, Department of Bioengineering, Houston, Texas; Dalhousie University, CANADA

## Abstract

The motor protein prestin is a member of the SLC26 family of anion antiporters and is essential to the electromotility of cochlear outer hair cells and for hearing. The only direct inhibitor of electromotility and the associated charge transfer is salicylate, possibly through direct interaction with an anion-binding site on prestin. In a screen to identify other inhibitors of prestin activity, we explored the effect of the non-steroid anti-inflammatory drug diflunisal, which is a derivative of salicylate. We recorded prestin activity by whole-cell patch clamping HEK cells transiently expressing prestin and mouse outer hair cells. We monitored the impact of diflunisal on the prestin-dependent non-linear capacitance and electromotility. We found that diflunisal triggers two prestin-associated effects: a chloride *independent* increase in the surface area and the specific capacitance of the membrane, and a chloride *dependent* inhibition of the charge transfer and the electromotility in outer hair cells. We conclude that diflunisal affects the cell membrane organization and inhibits prestin-associated charge transfer and electromotility at physiological chloride concentrations. The inhibitory effects on hair cell function are noteworthy given the proposed use of diflunisal to treat neurodegenerative diseases.

## Introduction

The cylindrically shaped, polarized epithelial cochlea outer hair cells (OHC) respond to changes in membrane potential. Hyperpolarization of the membrane voltage triggers an elongation of the OHC while depolarization triggers cell shortening [1,2]. This voltage-dependent motility enhances sound amplification in the cochlea [1] and the electromotility motor has been identified as the transmembrane protein prestin (SLC26A5) [3]. When present in the cytoplasmic membrane, prestin converts changes in the electrical field into mechanical force, without the use of ATP, calcium or any identified cytoskeletal protein [4]. OHC electromotility is associated with a nonlinear voltage-to-capacitance relationship that can be fitted to a two-state Boltzmann function. This non-linear capacitance (NLC) reflects the voltage-dependent charge movement that occurs within the membrane and is used to monitor prestin activity [3,5,6]. Despite an essential role in voltage sensing, the biophysical basis of the charge movement is uncertain. In the intrinsic voltage sensor model, the voltage-sensing depends on the movement of charged amino acids [7] while in the extrinsic voltage sensor model, intracellular anions such as chloride translocate through prestin in response to voltage [4]. Regardless, the modulation of the charge movement and of OHC electromotility by anions [4,8,9] supports the existence of a monovalent-anion binding site in prestin [4,7,10].

The only direct inhibitor of prestin function is salicylate, which inhibits the charge movement and the associated electromotility, putatively by competing with chloride for the anion-binding site in prestin [4,7,11]. By contrast, temperature [12], intracellular pressure [13], or molecules like cholesterol [14–16], chlorpromazine [17–19] and lipophilic ions [20] are hypothesized to trigger changes in membrane properties (curvature, thickness and mechanics) that result in modifications of prestin function. Changes in lipid-bilayer properties have been associated with the modulation of many membrane proteins [21].

In order to understand the physiological consequences of prestin modulation, we aimed at identifying more direct effectors and inhibitors of prestin activity. Based on the effective inhibition of salicylate, we have investigated the effect of the salicylate-derivative diflunisal (DFL) on mouse OHCs and on HEKs expressing prestin[14,15,22,23]. DFL was discovered in the 1980’s to have improved lipophilicity, increased anti-inflammatory and analgesic properties over salicylate [24]. Interestingly, diflunisal prevents amyloid fibril formation *in vitro*, and is being investigated as a treatment for familial amyloid polyneuropathy, a multisystem disorder resulting from the deposition of fibril aggregates in tissues [25–27].

## Materials and methods

The HEK 293 model system, validated for electrophysiological studies of prestin activities [15,28,29], is used to monitor prestin function after presentation of NSAIDs. The effect of chloride on the inhibition of prestin by DFL and the resulting impact on electromotility are further investigated using OHCs.

### Cell culture

Stable HEKs 293 expressing prestin [22] were grown at 37°C with 5% CO_2_ in DMEM (Cellgro) complemented with 10% Tet-System Approved FBS (Clontech) in the presence of penicillin, streptomycin, G418 and hygromycin (Invitrogen). Doxycycline (Clontech) was added to the growing media at a final concentration of 2 μg/ml to induce the expression of prestin-mGFP. The stable cell line expressing prestin was constructed as described previously [22].

### Electrophysiology and capacitance recordings

Pipettes were pulled from borosilicate capillary tubes 0.8–1.1 x 100mm (Kimble Chase- for HEKs) or Thin Wall Patch Glass (Warner Instrument–for OHCs) to obtain 2.5 to 3.5 MΩ openings. The electrophysiology was performed using an EPC10 plus amplifier (HEKA). The intracellular blocking solution (ICB) contains 130 mM CsCl, 2 mM MgCl2,10 mM EGTA and 10 mM HEPES. For low chloride experiment, the chloride was replaced by glutamate. The bath blocking solution (ECB) contains 99 mM NaCl, 20 mM TEA- Cl, 2 mM CoCl2, 1.47 mM MgCl2, 1 mM CaCl2, 10 mM HEPES. These solutions are both titrated to pH 7.2, and osmo-adjusted with dextrose to 320 mosM for HEKs and 300 mosM for OHCs. Only healthy, single HEK cells showing suitable GFP fluorescence were assayed. OHCs were used within 4 hours of animal death, and within 1 hour of being in the recording chamber at RT. All cells retained for analysis exhibited series resistance less than 10 MΩ and membrane resistance in excess of 1 GΩ for HEKs and 300MΩ for OHCs. The intracellular pressure was controlled with a High-Speed Pressure Clamp (ALA). The membrane capacitance (Cm) was determined using a phase-sensitive detector implemented in PatchMaster (HEKA) as described elsewhere [23]. Briefly, we applied an 800-Hz, 10-mV sine wave and measured the current response as DC holding potential was stepped in 2 mV increments (0.4mV/ms). The phase shift monitored between the output and the input signals allows for determination of the membrane capacitance. The resulting capacitance versus voltage curve is fitted to the first derivative of the two-state Boltzmann function:

$$C_{m}=\frac{Q_{\max}\left(\frac{ze}{kT}\right)}{\exp\left(\frac{ze}{kT}(V-V_{1/2}\right)\times {\left(1+\exp\left(\frac{ze}{kT}(V-V_{1/2})\right)\right)}^{2}}+C_{lin}$$ (1)

Q_max_ is the maximum nonlinear electric charge movement provided by all active prestin during transition, V_1/2_ is the voltage at which half-maximal charge transfer occurs and z is the valence of charge movement. C_lin_ is the linear capacitance. Since variation in cell size causes differences in the maximal charge transfer Q_max_, the charge movement is normalized to C_lin_. This parameter, designated as “charge density” can be interpreted as a quantitative measure of the amount of functional prestin in the membrane. The NLC yielded by the stable HEK cell-line used here averaged the following parameters (value ±SD): z = 0.79 ± 0.12; V_1/2_ = -68.56 ± 18.18 mV; C_lin_ = 20.9 ± 8.7 pF; Q_max_ = 0.13 ± 0.1 pC; CD (Q_max_/C_lin_) = 6.30 ± 3.30 fC/pF.

NLCs obtained from OHCs were fitted using the two-state C_SA_ model equation [29]:

$$C_{m}=\frac{Q_{\max}\left(\frac{ze}{kT}\right)}{\exp\left(\frac{ze}{kT}(V-V_{1/2}\right)\times {\left(1+\exp\left(\frac{ze}{kT}(V-V_{1/2})\right)\right)}^{2}}+\frac{\Delta C_{SA}}{{\left(1+\exp\left(\frac{ze}{kT}(V-V_{1/2})\right)\right)}^{-1}}+C_{lin}$$ (2)

For each cell successfully patched, three NLC curve were collected per condition tested and the parameters of the fits were averaged. Each experimental condition was tested 3 times or more on independent cells. The average values and standard deviation reported in the results were calculated based on these independent measurements.

### Diflunisal application

The diflunisal was from Sigma Aldrich. Solutions were made on the same day as the experiment in ECB before adjusting the pH and the osmolarity. The buffer in the recording chambers (~400 μL) was exchanged with 4 mL of DFL solution, using a gravity-driven perfusion system (~ 2 mL/min). This was followed by an incubation of 2 minutes. Chemicalize.org was used for pKa, logP and logD predictions [30]. The effect of perfusion alone was determined by measuring the changes occurring upon perfusion of clean extracellular buffer or buffer containing a higher NaCl concentration (an extra 10 mM, yielding a total 109 mM in the ECB). No significant impact on the NLC parameters was observed with these control experiments (S1 Fig).

### OHC isolation

One to 2 month old C57BL/6 mice were euthanized by CO_2_ asphyxiation, decapitated and dissected immediately. The cochlea was then separated from the temporal bone in ECB and cut in half. Each part was individually incubated in collagenase 0.5 mg/mL in ECB for 20 minutes at 37°C to dissociate the cells. The samples were then kept on ice until use. At the time of transfer to the electrophysiology chamber, the OHCs were further dissociated by gentle pipetting. Selected cells had a uniform cylindrical shape with a basally located nucleus and were used within 4 hours post-mortem. The Institutional Animal Care and Use Committee of Rice University and Baylor College of Medicine approved all experiments and procedures involving animals.

### Electromotility

Images were acquired with *μmanager* [31] controlling a a Retiga 2000R camera (Q-imaging), using a 63X objective on an Axiovert 200 microscope (Zeiss). Mice OHCs were imaged at 50 fps at a definition of 5.5 pixels/μm. The membrane surface area was calculated from the cell diameter, measured at the nucleus level, and the cell length, measured between the base and the apex (average A = 623±100 μm^2^ for n = 34 cells). Cell movement was analyzed with Video Spot Tracker (CCISMM), with trackers positioned at the base and the apex of the OHC. The distance between the base and the apex of the cell was plotted against the applied voltage. The resulting curve was fitted to a two-state Boltzmann equation:

$$L=\frac{L_{\max}}{1+\exp(-\alpha (V-V_{1/2}))}+L_{0}$$ (3)

## Results

### Effect of diflunisal on prestin in HEKs

**Fig 1:**
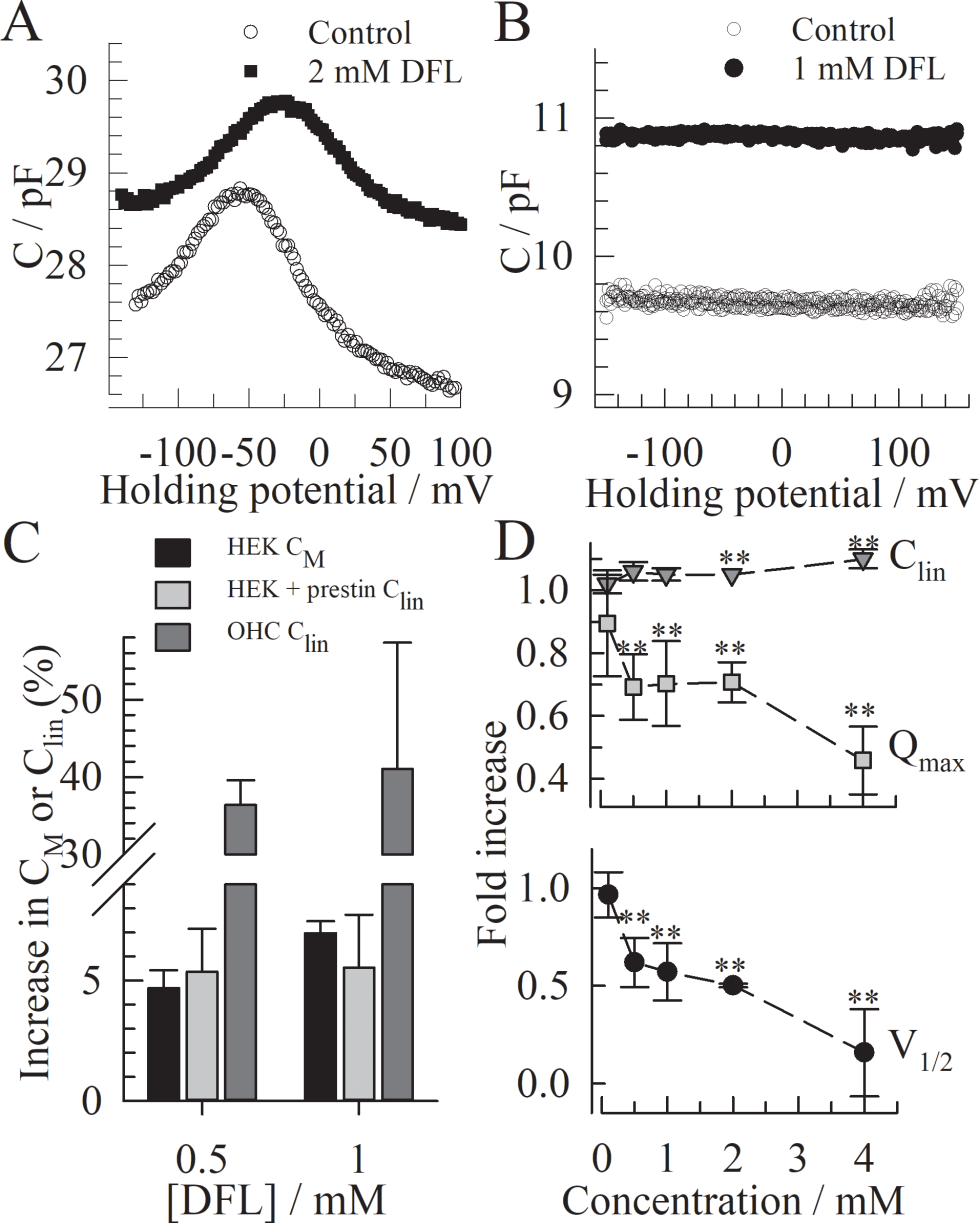
Effect of diflunisal on the NLC in HEKs expressing prestin. **(A)** Representative non-linear capacitance curves recorded on the same HEK cell expressing prestin, with (○) or without (●) DFL. **(B)**. Representative effect of DFL on the capacitance *vs*. voltage curve in the absence of prestin in HEKs. The membrane capacitance C_M_ of non-transfected HEKs was monitored at voltages from– 160 mV to +160 mV in the absence and presence of DFL. **(C)** Effect of DFL on the membrane capacitance in the presence (C_lin_) and absence (C_M_) of prestin. Both Clin and C_M_ were obtained from fits to the capacitance *vs*. voltage curves, using Eq 1 or a linear curve respectively. N = 4 for each condition. The error bars show the standard deviation. **(D)** Fold increase for each parameter obtained from fitting the NLCs to Eq 1; n≥3 cells for each condition. The error bar shows the standard deviation. The t-test is conducted between the values obtained upon perfusion of ECB with DFL versus ECB alone: * p<0.01; ** p<0.001.

The preliminary explorations of diflunisal (DFL) involved recording non-linear capacitance (NLC) in HEKs expressing prestin. For each cell, the NLC is recorded in the absence and in the presence of extracellular DFL and each NLC curve is fitted to Eq 1 (*Fig 1A*). DFL significantly affects all the measured parameters of the prestin-induced NLC. The V_1/2_ and charge density (Q_max_/C_lin_) are decreased by a DFL concentration as low as 100 μM and reaches significance above 500 μM. At 4 mM, the charge density is decreased by more than 50% and V_1/2_ is shifted by almost 70mV toward depolarized potentials. The apparent charge valence z is only significantly affected in the presence of 4 mM DFL (+11±4%). Additionally, the linear capacitance C_lin_ is increased by 5.3±1.7% at 0.5 mM and 10±4% at 2 mM, suggesting an effect of DFL on the membrane thickness or the dielectric constant. This could indicate partitioning of the molecules in the membrane despite the weak lipophilicity of DFL (logD_pH 7.2_ = 1 [30]). For this reason, the effect of DFL on the membrane specific capacitance C_M_ was further investigated in HEKs without prestin (*Fig 1B and 1C*). The membrane capacitance is increased in the presence of 0.5 and 1 mM DFL to a similar extend as C_lin_ is in HEKs with prestin. In the absence of prestin, DFL did not cause any voltage-dependent fluctuation of the capacitance between –200mV to +200 mV. We observe an almost full reversibility of the DFL-induced effect on the membrane or on prestin after washout of DFL with extracellular buffer (ECB). The partial inhibition of the charge transfer observed in HEKs encourages a more thorough investigation of the effect of DFL on prestin and on the outer hair cells (OHCs).

### Effect of DFL on outer hair cell function

**Fig 2:**
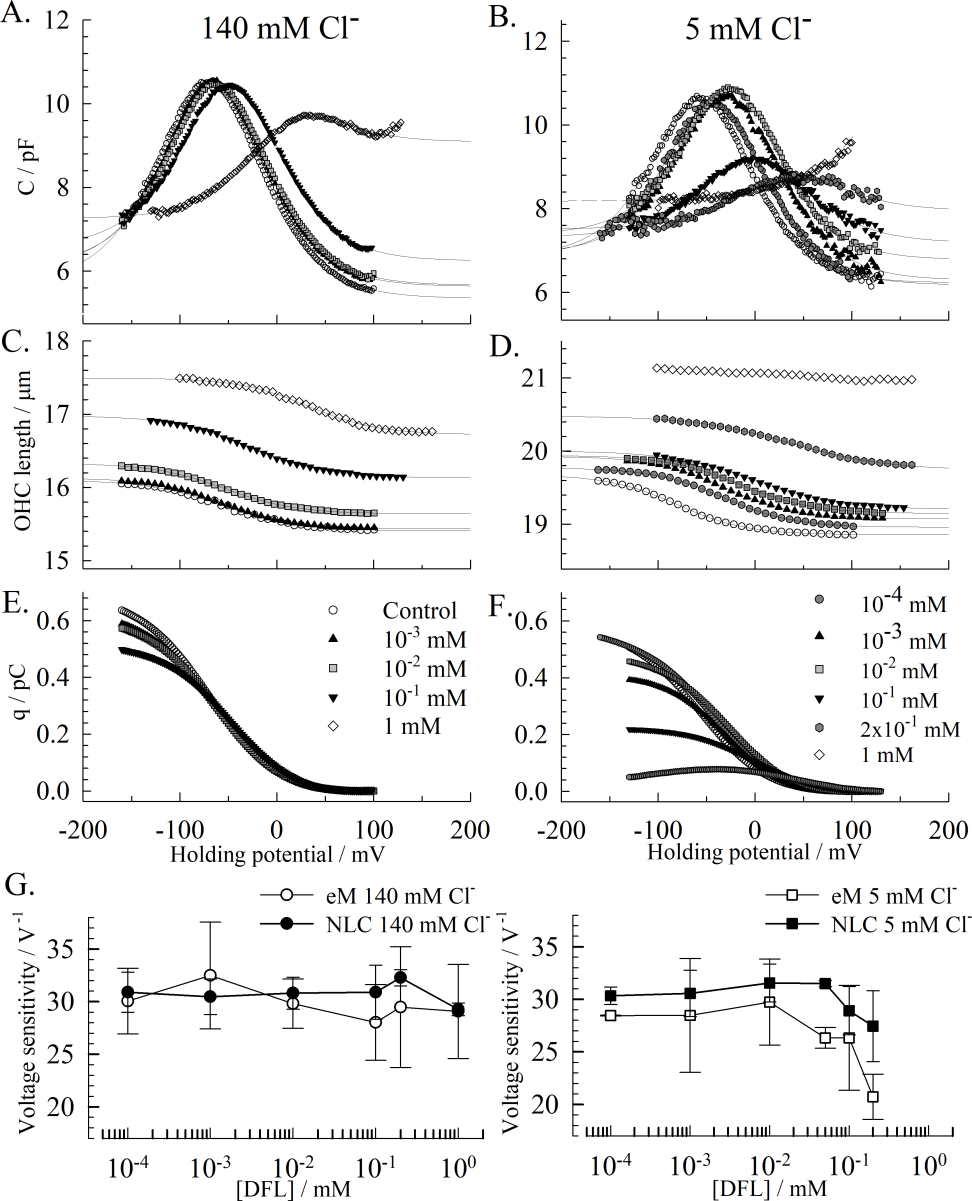
Effect of DFL on OHCs. The curves in A, C, E and in B, D F respectively are recorded on the same cells and in the same conditions, during hyperpolarizing voltage ramps. **(A) & (B)** Representative NLCs recorded on 2 different OHCs respectively with 140 mM and 5 mM chloride intracellularly. Increasing concentrations of DFL are perfused in the chamber, from 10^−4^ mM to 1 mM. The fits of the two-state C_SA_ model equation to the NLCs are shown as solid lines. In the absence of drug, the average parameters are: C_lin_ = 5.70±0.67 pF; C_max_ = 9.54±1.01 pF; Q_max_ = 0.47±0.07 pC; V_1/2_ = -42.4±12.9 mV; z = 0.84±0.4. **(C) & (D)** Cell length recorded concomitantly in μm. The solid lines are the resulting fits of the data to the two-state CSA model equation (Eq 2). In the absence of drug, the average characteristics of eM are L_0_ = 18±2.4 μm; L_max_ = 0.60±0.15 μm; V_1/2_ = -38.90±15.31 mV; α = -31.55±3.99 V^-1^. **(E) & (F)** Charge transfer against voltage, obtained from Q = Σ(ΔV.C). **(G)** The voltage sensitivity (*α = ze/kT*) for charge transfer and eM is determined at peak voltage and plotted against the DFL concentration. Each data point represents the average value from recordings on 3 cells or more, and the error bars are the standard deviation.

**Fig 3:**
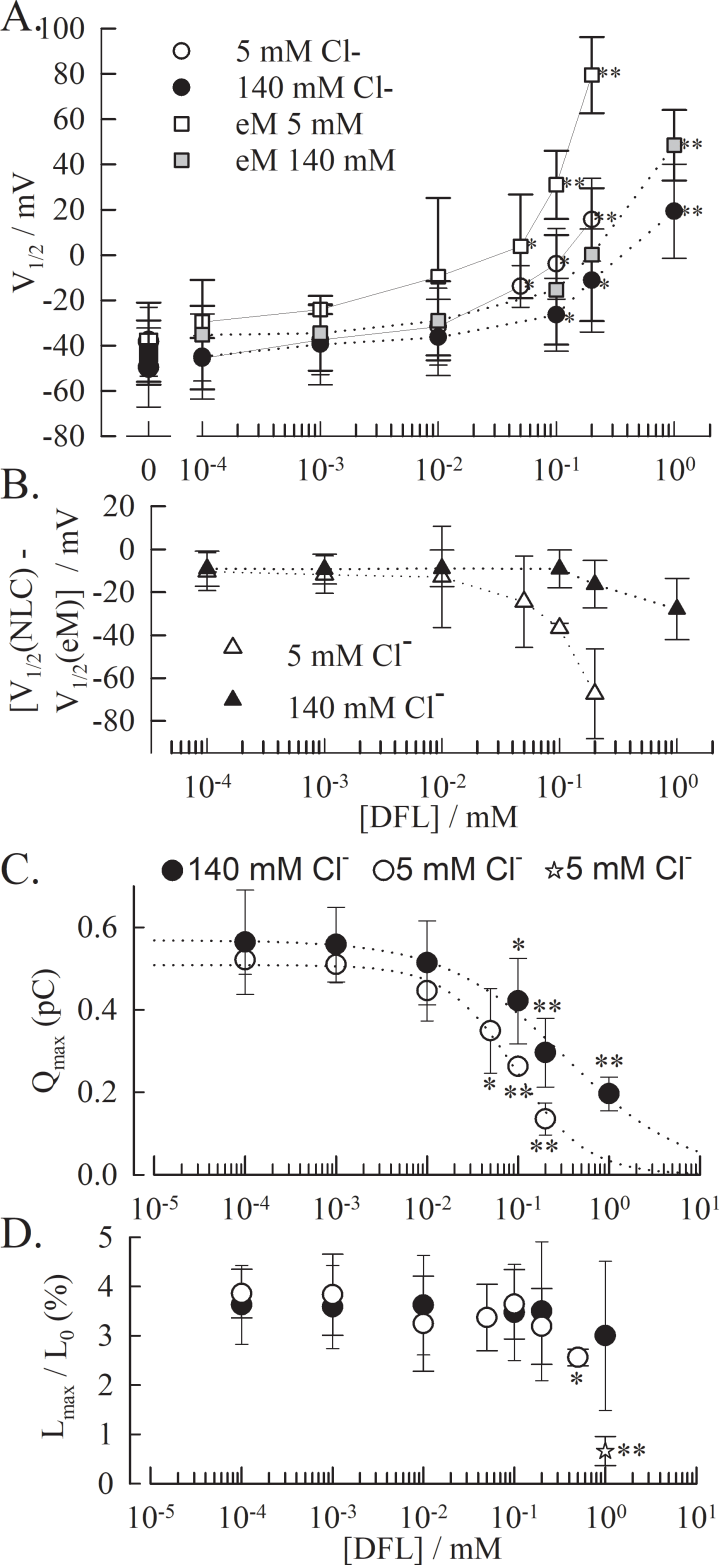
NLC and eM in the presence of DFL and in different chloride conditions. **(A)** V_1/2_ value obtained with OHCs from two-state Boltzmann fits of the NLC (○ at low & ● at high intracellular chloride) and the eM (◻ at low & ■ at high intracellular chloride), plotted against DFL concentration. **(B)** The difference V1/2(NLC)—V_1/2(eM)_ shows decoupling between charge transfer and electromotility as [DFL] increases. The t-test is conducted between the values obtained without DFL versus with DFL * p<0.01; ** p<0.001. **(C)** Inhibition of the NLC and **(D)** of the eM by DFL. The dotted line is the resulting fit of the data by a Hill equation (140mM Cl: n_H_ = -0.66, IC_50_ = 331 μM; 5mM Cl^-^: n_H_ = -1.12, IC_50_ = 98 μM), The resulting eM is only inhibited at concentrations above 0.5 mM in low chloride conditions. The value for eM at 1mM DFL at low chloride concentration was obtained from direct measurement of cell length at hyperpolarized and depolarized voltages (star). For all plots, the data points represent the average and the error bar is the standard deviation for n≥3 cells per condition.

We investigated the effect of DFL on OHCs that express endogenous prestin. Due to the reported prestin-salicylate sensitivity to chloride concentrations [32], we investigated the impact that high (140 mM) and low (5 mM) chloride concentrations have on the DFL inhibition of prestin. The effect of DFL on the NLC is similar to that described with HEK recordings (Fig 1) where the peak voltage is shifted to depolarizing potentials, the amplitude lowered and C_lin_ increased (Fig 2*A*). We also tracked the motility of the OHCs in response to voltage. Consistent with the shift in NLC and the increase in C_lin_, the electromotility (eM) curve is shifted to depolarized voltages and the total length of the cell is increased by DFL (Fig 2*C*). However, no apparent effect was noted on the amplitude of eM in high chloride conditions at the DFL concentration tested. DFL concentrations above 1mM weakened the pipette-OHC seal and could not be accurately assessed. However, lowering the chloride concentration enhanced the effect of the DFL on prestin, and yields a full inhibition of the NLC and eM at 1 mM in the –100 to + 160 mV voltage-range (Fig 2*B* and 2*D*). The dose-dependent inhibition of prestin by DFL is further observed on the charge-transfer during hyperpolarizing voltage ramps (Fig 3*E* and 3*F*). The maximum transfer significantly decreases with the DFL concentration, more so in low intracellular-chloride conditions.

Due to the asymmetry of the NLC, the dataset was fitted using the two-state C_SA_ model equation (Eq 2) [29]. In the presence of DFL, fits obtained using the two-Boltzmann function yielded an average root mean square error (RMSE) of 0.16 (± 0.07), while fitting with the C_SA_ model yielded a RMSE of 0.11 (± 0.06). The difference between the two fits was more apparent as the asymmetry of the NLC was reinforced in the presence of DFL. The presence of the extra component that tracks changes in specific membrane capacitance (C_SA_), fit the data more accurately, notably in hyperpolarizing conditions. Modifications in the rate of the charge movement and of the eM are evident and are extracted from the fit as *α = ze/kT*, also referred to as the voltage sensitivity. The resulting values are plotted in Fig 2*G* for each chloride condition.

A significant drop in the voltage sensitivity exists for both eM and NLC at DFL concentrations above 0.01 mM in low chloride conditions. At 0.2 mM DFL, the charge transfer rate drops to 28.9±2.3 V^-1^ for the NLC (from 33±1.2 V^-1^ w/o DFL) and to 25.32±4.3 V^-1^ for the eM (from 31.8±2.8 V^-1^ w/o DFL). Such a change in voltage sensitivity of the charge transfer has been reported in the presence of 10 mM salicylate, from 32.5 V^-1^ to 17.25 V^-1^ for *guinea pig* OHCs [29]. The parameters of the NLC and the eM affected by DFL in high (140 mM) and low (5 mM) intracellular chloride conditions were determined next. V_1/2_ was calculated for eM and NLC for each condition, and plotted against the concentration of DFL (Fig 3*A*). At 140 mM chloride, the shift in eM and NLC are similar, and significant at 0.1 mM DFL and above. The shift is significant at 0.05 mM DFL and above when in low chloride conditions, and is stronger for the eM than for the NLC. Such disparity between the V_1/2_(eM) and the V_1/2_(NLC) has previously been reported for low chloride conditions [33]. Notably, this disparity is amplified by the presence of DFL (Fig 3*B*). As described in the HEK cell model, the maximum non-linear charge movement Q_max_ decreases, in a DFL dose dependent manner, starting at concentrations as low as 0.01mM DFL (Figs 2*E*, 2*F* and 3*C*). The inhibition is greater at lower chloride concentrations, which approximate the *in vivo* condition [9]. In high and low chloride conditions, respectively, Q_max_ was decreased to 38% (1 mM) and 29% (0.5 mM) of the values recorded in the absence of DFL. Above these concentrations, the NLC could not be fitted accurately. These inhibition curves were fitted to a Hill equation to produce apparent IC_50_ of 331 μM and 98 μM in high and low chloride conditions, respectively (with Hill coefficient of -0.66 and -1.12, respectively).

The amplitude of the eM (plotted as % change in total cell length; Fig 3*D*), evidently resulting from the charge movement, is only inhibited at 0.5 mM DFL in low chloride conditions (p<0.01) and has an apparent full inhibition at 1mM (within the tested voltage range), which approximates the absence of NLC illustrated in Fig 2*B*. There was no significant decrease in eM amplitude at any concentrations at high chloride conditions. An identical disparity between the maximal charge movement Q_max_ and the maximal amplitude of the cell movement eM_max_ had been reported for decreased chloride concentrations [33], giving support to the *meno presto* model to explain the kinetic behavior of prestin. The full eM inhibition observed in the tested voltage-range at low chloride concentration and in the presence of 1mM DFL (Fig 2*D*) did not fit Eq 3. Therefore, the data point (star in Fig 3*D*) is calculated as the length change observed between the depolarized and hyperpolarized states in the voltage range, and not from a plateaued state (as observed in Fig 2*D* at 1 mM DFL).

**Fig 4:**
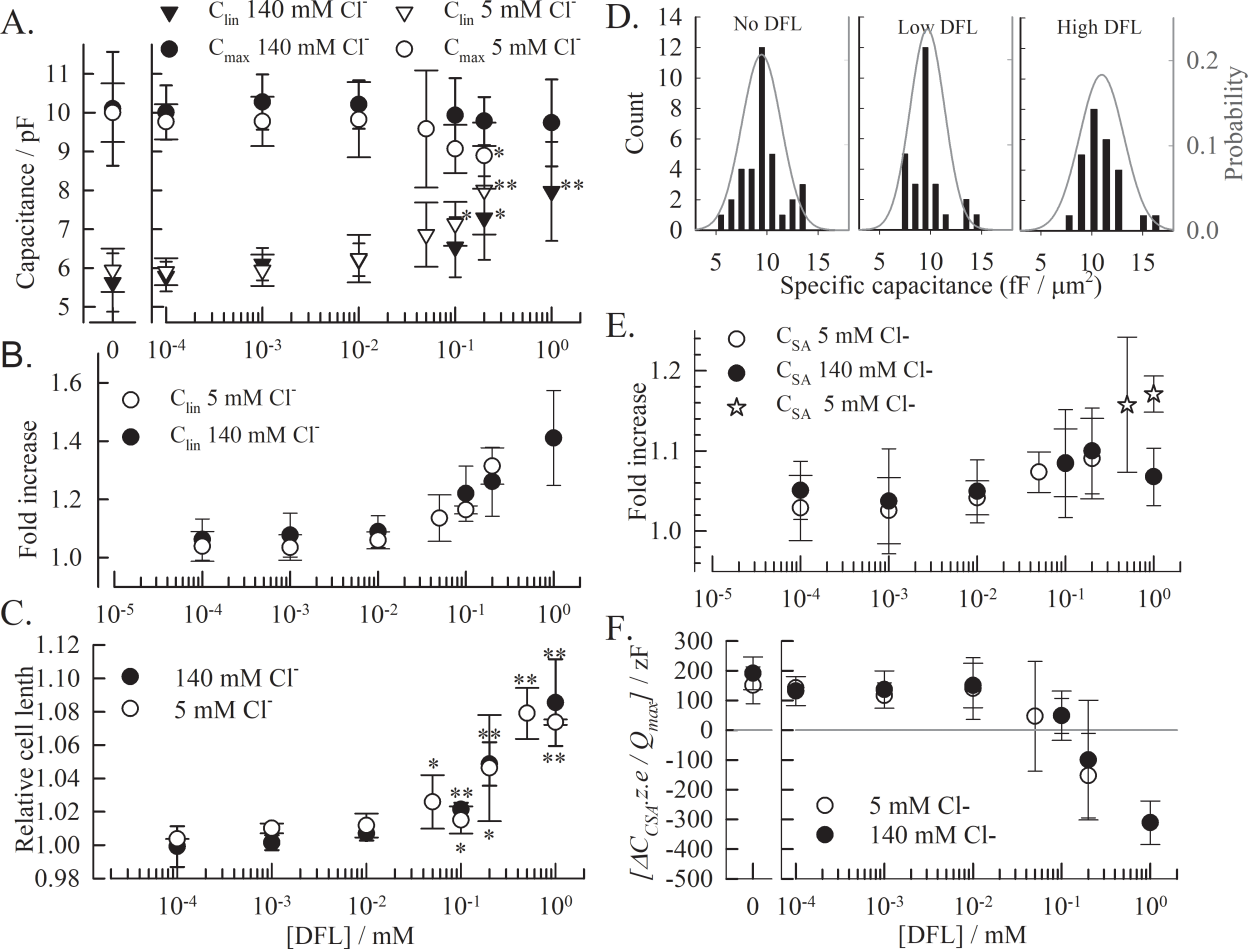
DFL affects the capacitance and the length of the OHC. **(A)** The effect of DFL on absolute C_lin_ and C_max_ values and **(B)** relative C_lin_ (compiled on a cell to cell basis) are compared to **(C)** the change in total cell length, plotted as the ratio of OHC length with DFL to without. In order to have reliable data at high DFL concentrations, we used the fully extended length to calculate this ratio. **(D)** Specific capacitance C_lin_/A (pF/μm^2^) is calculated for each OHC and showed in 3 distribution histograms. The diameter and length of each cell was measured and the resulting area was determined. Low DFL: [DFL]≤10^−2^ mM & High DFL: [DFL]>10^−2^ mM. The averages obtained from the probability distribution fitting of the raw data (grey line) are 9.45±1.9, 9.68±1.7 and 10.97±2.1 fF/μm^2^. (**E**) The surface area specific capacitance C_SA_ as well as **(F)** the difference between C_SA_ and C_lin_ (ΔC_SA_) are obtained from fitting the NLCs. ΔC_SA_ is normalized by dividing by the number of active motors *N* in the absence of drug on a cell to cell basis. Each value is averaged from 3 independent recordings or more, and the error bars represent the standard deviation. (t-test: * p<0.01; ** p<0.001).

The expanded OHC total length L_OHC_ also increases up to 8% with 1 mM DFL (Fig 4*C*). The effect is similar at high and low chloride concentrations, suggesting that it results from the partition of DFL in the membrane and not from the interaction with prestin. However, there is no linear correlation between the NLC parameter C_lin_ and the increase in L_OHC_. The 8% change in cell length corresponds to an increase of ~40% in C_lin_ (Fig 4*A*). The specific capacitance for the OHCs was determined for each cell and condition as C_lin_/A (pF/μm^2^), where the area *A* was calculated using the compacted length of the OHC and the diameter at the nucleus level. Histograms of the specific capacitance as well as the probability distribution fitting to the corresponding raw data are plotted in Fig 4*D* [34]. We regrouped the cells into three groups based on the impact of DFL on C_lin_ and cell length (Fig 4*A*–4*C*): no DFL; [DFL]≦10^−2^ mM where neither C_lin_ or length are modified and [DFL]>10^−2^ mM where C_lin_ and cell length are significantly altered. The data obtained in the presence of 1mM DFL were not used since precise C_lin_ were difficult to obtain. The specific capacitance increases in the presence of DFL from 9.45 fF/μm^2^ to 10.97 fF/μm^2^, whereas, the low DFL concentrations had no significant impact (9.68 fF/μm^2^). These values are comparable to the specific capacitance of 10±1.7 fF/μm^2^ reported for guinea pig OHCs [34].

Finally, we observed modifications of C_SA_ (Fig 4*E*), the membrane specific capacitance, which has been associated with changes in surface area during eM [8,29,33]. We investigated the effect of DFL on ΔC_SA_ (which is the change in capacitance, in regard to C_lin_) that occurs when prestin goes from a compacted (depolarized) to expanded (hyperpolarized) state. The behavior of ΔC_SA_ is independent of the chloride concentration. In the absence of DFL, ΔC_SA_ = 0.59 ± 0.24 pF (5 mM Cl^-^) and 0.64 ± 0.22 pF (140 mM Cl^-^). Since the number of motors in the membrane is N = Q_max_/*z*.*e*, the normalized corresponding value for each motor δC_SA_ is *ΔC*_*SA*_.*z*.*e/Q*_*max*_, i.e. 151±62 zF and 191±55 zF in the absence of DFL at 5 mM and 140 mM chloride, respectively. The resulting values are plotted against the DFL concentration in Fig 4*F*. At lower concentrations, δC_SA_ is not affected, but it decreases at concentrations above 10^−2^ mM, and reaches negative values. Such a drop in δC_SA_ is also observed with increasing concentrations of salicylate, but does not go lower than the value measured in the absence of drug [8]. However, the effect of salicylate was not accompanied by such an increase in C_lin_. If the value of C_SA_ measured at hyperpolarized voltages (C_lin_- ΔC_SA_) is plotted against the concentration of DFL we observe a slight increase of C_SA_ at inhibiting concentrations of DFL in low chloride conditions (Fig 4*E*). The constant C_SA_ at lower DFL concentration implies that the prestin-area related capacitance is not affected. The capacitance *vs* voltage data for 0.5 and 1 mM DFL at 5mM chloride could not be fitted to the two-state C_SA_ model equation, due to the shift of the NLC and the full inhibition of the NLC.

## Discussion

### Diflunisal inhibition compared to other prestin modulators

Diflunisal is an inhibitor of the prestin NLC at low intracellular chloride (1mM DFL, 5mM Cl^-^) and results in the disruption of OHCs electromotility. Sub-inhibiting concentrations of DFL (< 0.5mM) affects the charge transfer properties of prestin, lowering the Q_max_ and shifting V_1/2_, without diminishing the amplitude of electromotility. This decoupling between electromotility and charge transfer which has been described at low chloride concentrations, and described by the *meno presto* model, is amplified here by DFL [33]. In the *meno presto* kinetic model, which integrates the two-state C_SA_ model used in our analysis, the presence of slow intermediate steps between chloride binding and the two-state Boltzmann voltage-dependent process predict a chloride-dependent disparity between the charge movement and the motor activity of prestin. The differences between the V_1/2_ values (Fig 3B) or between the amplitudes (Q_max_ *vs*. L_max_; Fig 3C and 3D) observed in the presence of DFL are compatible with this model and with a direct competition of DFL with chloride [32,33]. Moreover, the effect of DFL on the membrane highlight that even when elongated by ~5% (4.9±1.3% and 4.6±3.1% in high and low chloride conditions, respectively; Fig 4C) the OHCs are still able to yield an electromotive response with an amplitude > 3% (3.5±1.4% and 3.1±0.8% at high and low chloride, respectively; Fig 3*D*).

Prestin can also be inhibited by increased membrane tension, increased temperature and by application of salicylate. DFL induces a simultaneous decrease of Q_max_ and a positive shift of V_1/2,_ which is similar to the impact of increased membrane tension [13,35] and increased temperature [12]. This dual impact is also observed when intracellular chloride is decreased [36], hinting at the possibility that DFL could be competing with chloride ions. This is further supported by the chloride-dependency of this inhibition, with DFL having a stronger effect at low chloride concentrations. As reported for salicylate, this suggests that DFL competes with chloride ions [4,9], or modifies the interaction of prestin with chloride, which is essential for prestin function (as discussed in [7]). The effect of DFL is comparable to the effect of salicylate at 5 mM chloride (IC_50SAL_ = 79.8 μM on *guinea pig* OHC *vs* IC_50DFL_ = 98 μM), but is greater at 140 mM chloride (IC_50SAL_ = 964 μM on *guinea pig* OHC IC_50DFL_ = 331 μM)[9]. If we consider a competitive inhibition mechanism, DFL is more able to displace Cl^-^ ions than salicylate and therefore has a lower K_i_. This is surprising, considering both molecules have the same single negative charge, but that DFL has an extra aromatic ring. The greater steric occupancy of diflunisal (198 Å^3^ van der Waals volume *vs*. 118Å^3^ for salicylate [30]) should impair access to the binding site. This implies that the binding site for chloride, and salicylate and DFL, is not restrictive but accessible to larger molecules.

Finally, the blood-cochlear barrier is physiologically similar to the blood-brain barrier [37], and DFL, like salicylate, has a polar surface area (PSA = 60.4 A^2^) low enough to allow these molecules to penetrate the blood-cochlear barrier [38,39]. Therefore, the effect of diflunisal on isolated OHCs can have physiological consequences similar to the salicylate effect on tinnitus [40].

### Diflunisal effects on the lipid bilayer

DFL provokes a chloride-independent increase in membrane surface area and specific capacitance greater than that observed for salicylate (Fig 4). This substantial effect of DFL on membrane properties is consistent with a higher affinity of DFL for a hydrophobic environment, and is accompanied by a chloride-independent increase in C_lin_. The interaction of DFL with the membrane is illustrated by the dramatic increase in OHC area (35.9 ± 17.7, 54.6 ± 17.4 and 53.3 ± 29.7 μm^2^ for 0.2, 0.5 and 1.0 mM DFL, respectively). Assuming a steric effect, and considering a projection area between 33.8 and 70.6 Å^2^ for DFL [30], the area change corresponds to the partition of 7.8x10^6^ to 16.5x10^6^ molecules of DFL in the membrane at 0.5mM DFL. This is more than the ~4x10^6^ DFL molecules predicted by the logD_DFL_ of 0.41 [30], suggesting that the elongation of the OHC by DFL is not solely due to the spatial occupancy of DFL in the membrane. The OHC lateral wall is constituted of a corrugated plasma membrane [41–45] which is modeled to anchor to the cytoskeleton at regular intervals [46]. Modifications of the curvature of this local bending of the membrane have significant impact on prestin. The amphipaths CPZ^+^ and TNP^-^ localize to the inner and outer leaflet to cause local inward and outward bending of these corrugations, respectively, and both result in a depolarized shift of the NLC [11,17,18,47]. Similarly, the preferential partitioning of DFL^-^ in the outer or inner leaflet of the membrane could modify the radius of the local membrane curvature and in turn further affect the apparent OHC length as well as V_1/2_.

Moreover, the presence of DFL in the membrane increases the specific membrane capacitance from 9.45 to 10.97 fF/μm^2^ (Fig 4*D*), suggesting an additional effect of DFL on the electrical properties of the membrane. Membrane capacitance depends on the membrane area (*A*), the membrane thickness (*d*) and the dielectric constant of the membrane (*ε*) where *C*_*m*_ *= ε*.*A/d* [48]. Consequently, the increase of the specific membrane capacitance demonstrates a decrease in membrane thickness *d* or an increase of the membrane dielectric constant *ε* or both. Interestingly, changes in membrane thickness have also shown to impact prestin. Decreasing membrane thickness by adding PC12:0 increases C_lin_ by 20% and shifts V_1/2_ to depolarized values. However, no change in Q_max_ has been observed upon decrease in membrane thickness [49]. DFL might therefore have two effects: *(i)* it impacts C_lin_ and V_1/2_ when partitioning in the membrane (see Fig 3A, the shift in NLC is not dependent on chloride concentration), and *(ii)* it inhibits charge transfer when interacting with prestin.

### Diflunisal affects OHCs and HEKs differently

The effects of DFL on the NLC and on C_M_ are more effective in native OHCs than in HEKs. At 0.1 mM DFL in HEK, prestin is barely affected, with V_1/2_ shifting 1.4 mV and Q_max_ dropping by 10%, while at the same 0.1 mM DFL in OHC, the V_1/2_ shifts 18 mV and Q_max_ is lowered by 25%. Such a difference is observable for all concentrations tested on both HEKs and OHCs (<1 mM). Salicylate is also more effective on OHCs, compared to HEKs expressing prestin. The IC_50_s estimated from published data with OHCs [50] and HEKs expressing rat prestin[4] were 220 μM and 640 μM, respectively, both with 140 mM intracellular chloride. In our experiments, for the same conditions, the apparent IC_50_ from a Hill fit to Q_max_ *vs*. [DFL] is 331μM for OHCs and 2.76 mM for HEKs at 140 mM intracellular chloride. Finally, the effect on membrane capacitance is more pronounced in OHCs than in HEKs, with an increase of C_lin_ by 2% in HEKs vs. 22% in OHCs at 0.1 mM DFL (and 5% vs. 40% at 1 mM DFL). The different lipid compositions in OHCs and HEKs can alter the interaction of the negatively charged DFL with the membrane and the subsequent partitioning of the molecules. Moreover, the density of prestin in the OHC membrane is very high and therefore has an impact on the dielectric constant [51] and the fluidity of the membrane, which could be affected differently by DFL. Importantly, the strong and specific interaction of DFL with hearing cells will lead to more specific damage to the cochlea.

### Diflunisal and the surface area capacitance C_SA_

DFL has a greater effect on C_lin_ than on the surface area capacitance measured at hyperpolarized voltages (C_SA_). C_SA_ illustrates the increase in membrane capacitance due to the change in prestin-dependent membrane surface area, and not to the charge transfer_._ The increase in C_SA_ observed at inhibiting conditions (Fig 4*E*) is consistent with prestin being maintained in an expanded state in the presence of DFL, similarly to what has been reported for salicylate [8]. Similarly, the drop in δC_SA_ (Fig 4*F*) reflects the decrease in the number of functional motors due to the presence of the inhibitor, as reported for salicylate ([32]).

The value of the C_SA_ / area (A) relationship does not significantly change in the absence (8.8±1.8 fF/μm^2^) and presence (9.82 ±1.5 fF/μm^2^) of DFL (data determined for C_SA_/A as for C_lin_/A in Fig 4*D*). Even when the NLC is inhibited at 1 mM DFL in low chloride conditions, the measured specific C_SA_ is 10.9±2.2 fF/μm^2^ (n = 4). This discrepancy between the capacitance at depolarizing and hyperpolarizing membrane potentials could stem from a voltage dependent repositioning of the charged DFL^-^ molecule in the membrane, resulting in a decrease of *d* or an increase of *ε* at depolarizing but not hyperpolarizing potentials. DFL showed no voltage-dependent effect on the capacitance of HEKs without prestin, implying that either prestin or the OHC corrugations are necessary for these DFL-induced membrane modifications.

## Conclusion

Although diflunisal is a commonly used NSAID, the specific effects of diflunisal on hearing have not been well studied. We have investigated how diflunisal affects the function of outer hair cells and its effect on the motor protein prestin. Despite being a derivative of salicylate with identical charge, diflunisal has a lower IC_50_ at high chloride concentration and a similar one at low chloride concentration. It triggers a greater elongation of the OHC and initiates a greater increase in membrane capacitance (C_lin_ and C_SA_) than does salicylate. Notably, this study revealed clues about the biophysical mechanisms of action on prestin. We demonstrate that the impact of diflunisal on prestin involves a dual mechanism: *(i)* diflunisal directly interacts with prestin through competition with chloride ions and *(ii)* modifies the properties of the membrane, which in turn affect the NLC [4,7,11]. Consequently, if the competition with chloride is specific to prestin, DFL might affect other mechano-sensitive membrane proteins through alteration of membrane material properties [26].

Finally, diflunisal has promising therapeutic properties–in preventing the formation of amyloid fibrils [26], as an anti-tumor agent against cancerous cell growth [52], and as an osteoprotectant in *Staphylococcus aureus*-induced osteomyelitis [53]. These recent attempts to repurpose diflunisal highlights the necessity to further investigate the impact of this molecule on hearing to understand the potential of irreversible side effects.

## Supporting information

S1 Fig Perfusion control—In order to test the effect of perfusion alone, and to control the impact of extra Na+ and Cl- that are added with some NSAIDs salts, NLCs were recorded on HEKs expressing prestin before and after the perfusion of regular ECB (clear bars) or ECB containing 109 mM NaCl (i.e. 10 mM extra NaCl–dark bars). The bars show the average change for each parameter ± standard deviation. No statistically significant difference is observed.
(TIF)

## Abbreviations

DFL: diflunisal
ECB: extracellular buffer
HEK: human embryonic kidney
ICB: intracellular buffer
NLC: non-linear capacitance
NSAID: non-steroid anti-inflammatory drugs
OHC: outer-hair cell
C_lin_: linear capacitance
C_sa_: surface area capacitance
eM: electromotility
