# Supplementary figures and images for: Diflunisal inhibits prestin by chloride-dependent mechanism
Source: PLoS One. 2017 Aug 17;12(8):e0183046. doi: 10.1371/journal.pone.0183046 (PMC5560734; doi:10.1371/journal.pone.0183046)

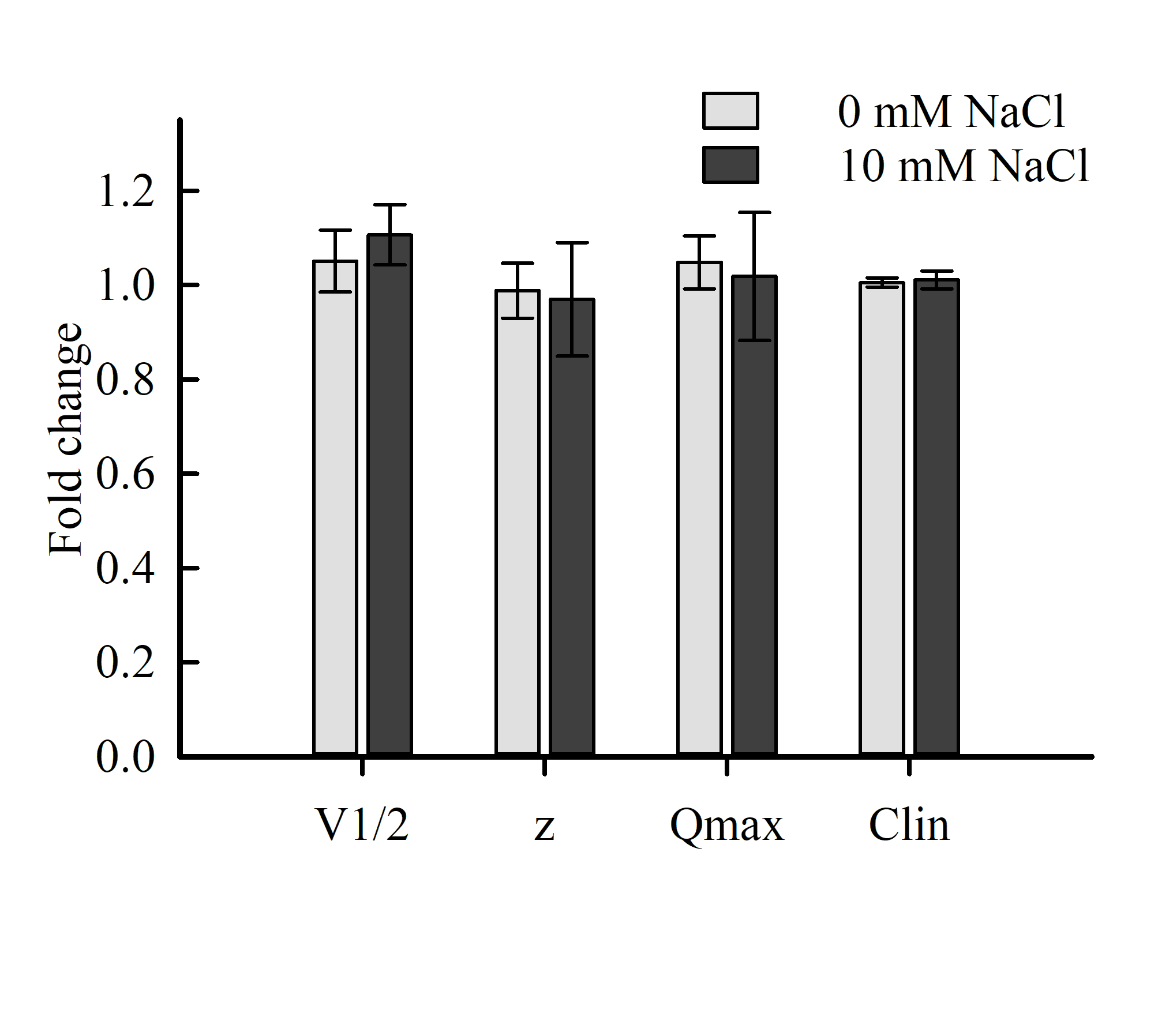

Supplement: S1 Fig — The bars show the average change for each parameter ± standard deviation. No statistically significant difference is observed. (TIF) [file pone.0183046.s001.tif]
